# Dendritic Targeting in the Leg Neuropil of *Drosophila*: The Role of Midline Signalling Molecules in Generating a Myotopic Map

**DOI:** 10.1371/journal.pbio.1000199

**Published:** 2009-09-22

**Authors:** David J. Brierley, Eric Blanc, O. Venkateswara Reddy, K. VijayRaghavan, Darren W. Williams

**Affiliations:** 1 Medical Research Council (MRC) Centre for Developmental Neurobiology, King's College London, London, United Kingdom; 2 National Centre for Biological Sciences, Tata Institute of Fundamental Research, Bangalore, India; University of Washington, United States of America

## Abstract

Neural maps are emergent, highly ordered structures that are essential for organizing and presenting synaptic information. Within the embryonic nervous system of *Drosophila* motoneuron dendrites are organized topographically as a myotopic map that reflects their pattern of innervation in the muscle field. Here we reveal that this fundamental organizational principle exists in adult *Drosophila*, where the dendrites of leg motoneurons also generate a myotopic map. A single postembryonic neuroblast sequentially generates different leg motoneuron subtypes, starting with those innervating proximal targets and medial neuropil regions and producing progeny that innervate distal muscle targets and lateral neuropil later in the lineage. Thus the cellular distinctions in peripheral targets and central dendritic domains, which make up the myotopic map, are linked to the birth-order of these motoneurons. Our developmental analysis of dendrite growth reveals that this myotopic map is generated by targeting. We demonstrate that the medio-lateral positioning of motoneuron dendrites in the leg neuropil is controlled by the midline signalling systems Slit-Robo and Netrin-Fra. These results reveal that dendritic targeting plays a major role in the formation of myotopic maps and suggests that the coordinate spatial control of both pre- and postsynaptic elements by global neuropilar signals may be an important mechanism for establishing the specificity of synaptic connections.

## Abstract 2

During development of the *Drosophila* motor system, global guidance cues control and coordinate the targeting of both input and output elements of the neural system.

## Author Summary

During development the axons of sensory neurons generate highly ordered ”sensory maps„ within the nervous system that represent specific qualities of the environment. Much less is known about the anatomical organization and development of motor systems. Here, we show that the leg motoneurons of *Drosophila* organize their dendrites within the central nervous system in a way that reflects the position of the muscles they innervate. These motoneurons generate a ‘myotopic map’ by targeting the growth of their dendrites (sites of synaptic input) into discrete territories during development. The precise targeting of dendrites along the mediolateral axis is controlled by the signaling molecules Slit and Netrin, which are secreted by midline cells. These proteins act as global guidance cues and exert their effects via distinct signaling pathways using receptors called Roundabout and Frazzled, respectively. Previous studies have shown that Slit also helps to position the termini of axons (sites of synaptic output), independent of their synaptic partners. We suggest that the coordinated targeting of both input and output elements of a neural system into a common space using shared global guidance cues could be a simple way of establishing the specificity of synaptic connections within neural networks.

## Introduction

The fidelity with which connections are made between neurons is a striking feature of nervous system design and essential for proper function [1]. How appropriate presynaptic and postsynaptic elements are brought together during development to generate such ordered connectivity is still a major unanswered question in neurobiology [2].

Most developmental studies investigating the generation of neural maps [3] or synaptic laminae [4] have focussed on the role that presynaptic elements play in establishing normal connectivity and the mechanisms that guide axons [5]. This “axonocentric” bias is understandable as the orderly growth of axons to their targets often reveals an explicit anatomical framework, upon which one can ask questions about mechanisms of network formation [6]. The role that dendrites, the major postsynaptic elements, play in the development of connectivity has been much less explored [7]. Dendrite shape is known to have important implications for neuron function as it determines a cell's integrative properties [8] and dictates the synaptic inputs it will receive [9],[10]. Thus cell-type-specific programs of dendrite development ultimately have a profound effect on the role a cell plays within a network [11]. Two very different modes of growth can generate a dendritic tree of the same basic shape: neurons can either profusely elaborate dendrites across a wide field and then selectively remove branches from inappropriate territories, or alternatively, dendrite growth can be targeted into distinct territories using guidance mechanisms similar to those found in axons [12]. Examples of both types of growth have been observed. The first mode of growth is seen in mammalian retinal ganglion cells to generate ON and OFF sub-laminae of the Inner Plexiform Layer [13]. The second mode of growth, “dendritic targeting”, is seen in the generation of both neural maps and synaptic laminae. In *Drosophila*, second-order projection neurons, which convey olfactory information to higher brain centres, target their dendrites to form a “protomap” prior to the arrival of presynaptic olfactory receptor neurons [14] suggesting targeted outgrowth. Similarly, imaging studies in zebrafish show that retinal ganglion cells target the growth of their dendrites to generate lamina-specific projections patterns [15].

Our understanding of neural network structure and function has benefited greatly from studies on the sensorimotor systems of vertebrates [16] and invertebrates [17]. Recent data reveal that the embryonic motoneurons of *Drosophila* generate a dendritic map within the CNS that represents the innervation of body wall muscles [18]. These central projections are highly ordered and likely reflect some underlying organization of pre-motor interneurons within the network. The map develops in the absence of target muscles, glial cells, or competitive interactions with adjacent dendrites, suggesting that coordinated cell-intrinsic programs for targeting are likely to be important for its assembly [18].

Although our understanding of the molecular mechanisms that control dendritogenesis is still incomplete, a number of transcription factors have been identified that coordinate the patterning of dendritic maps [19],[20],[21]. At present however the only downstream “effector” molecule known to be required for dendritic map development is Semaphorin-1a. Both loss- and gain-of-function experiments demonstrate that the levels of Semaphorin-1a, acting cell-autonomously as a receptor or part of a receptor complex, direct the dendritic targeting of projection neurons along the dorsolateral to ventromedial axis of the antennal lobe during map formation [22].

Here we investigate how the dendrites of leg motoneurons are targeted to distinct neuropil territories and how these mechanisms can collectively generate a neural map. The majority of leg motoneurons are born during larval life and the bulk of those are derived from a single neuroblast lineage, lineage 15 [23, unpublished data]. The neurons of lineage 15 form stereotyped projection patterns, dependent on their birth-order within the lineage. Early-born cells innervate proximal muscle targets and elaborate dendrites from medial to lateral territories, whereas late-born cells innervate more distal muscle groups within the leg and establish dendritic arborizations that are largely confined to lateral territories in the neuropil. Here we show how two subtypes, within this lineage, generate their distinct dendritic arborizations by targeting growth into specific territories using the midline signalling systems of Slit-Robo and Netrin-Fra. These data suggest that cell intrinsic blends of guidance molecules marshal the dendrites of this lineage into appropriate territories in a coordinated fashion to generate a myotopic map. Previous studies in *Drosophila* have revealed that both sensory neurons and interneurons position their synaptic terminals using the midline signalling cues [24],[25]. We propose that during development the targeting of both pre- and postsynaptic elements into the same space using global, third-party guidance signals could provide a simple way of establishing the specificity of synaptic connections.

## Results

### Birth-Order Dependent Projections of a Neuroblast Lineage Generate a Myotopic Map

To identify the axonal and dendritic projection patterns of leg motoneurons, we performed MARCM analysis [26] with the OK371-GAL4 driver line, which robustly labels motoneurons [27]. Our previous work revealed that a single postembryonic neuroblast (insect neural precursor cell) generates a lineage containing solely motoneurons (lineage 15) [23]. Most neuroblasts in *Drosophila* divide asymmetrically to generate themselves and a ganglion mother cell, which in turn divide once to give two neurons. With MARCM analysis the precise timing of clone induction by heatshock allows “snapshots” of the type of neuron born at different points within a lineage.

**Figure 1:**
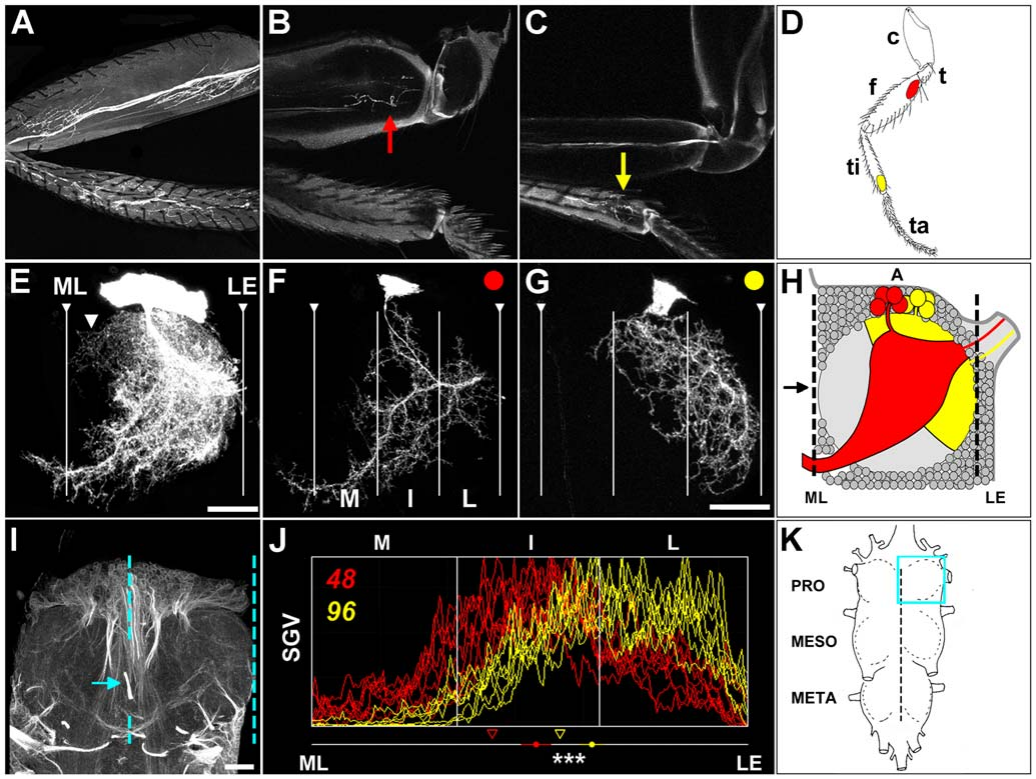
Birth-order dependent projections of lineage 15 in adult *Drosophila*. (A) Axonal projections of neuroblast clone of lineage 15 motoneurons revealing the three areas innervated in the prothoracic leg. (B) Motoneuron born following heatshock at 48 h AH innervates the pre-tarsal flexor muscle group located in the proximal femur (red arrow). (C) Motoneuron born following heatshock at 96 h AH innervates muscle groups located in the distal tibia (yellow arrow). (D) Cartoon showing segments of prothoracic leg including coxa (c), trochanter (t), femur (f), tibia (ti), and tarsi (ta) and the position of muscle groups innervated by motoneurons born at 48 (red) and 96 h AH (yellow). (E) The central projections of a neuroblast clone of lineage 15. Cell bodies located in a superficial cellular cortex surrounding a dense fibrous neuropil. The primary neurites of lineage 15 enter the anterior cortex and arborize extensively throughout the neuropil. In the anterior leg neuropil dendrites cover lateral territories extending a few processes towards medial territories (arrowhead); in the posterior neuropil, large dendritic branches extend towards and cross the midline and project into the contralateral leg neuropil. The midline (ML) and the lateral edge (LE) were determined by neuroglian staining. Scale bar = 20 µm. (F) Motoneurons born at 48 h AH elaborate their dendrites in medial (M), intermediate (I), and lateral (L) territories. A major posterior branch projects crosses the midline into the contralateral leg neuropil. (G) The dendrites of motoneurons born at 96 h AH are restricted to the intermediate and lateral territories of the leg neuropil. (H) Cartoon showing the organization of the central projections of two leg motoneurons in the prothoracic neuromere. The dendritic fields of 48 h (red) and 96 h AH (yellow) motoneurons within leg neuropil (light gray) surrounded by a cellular cortex (circles). A refers to anterior. Arrow shows the position of paired bundles of lineage 2. (I) Anti-neuroglian staining reveals bundles of adult specific lineages (arrow) and fibrous neuropil in the prothoracic neuromere. The lines indicate the midline and the lateral edge of the leg neuropil in the right hemineuromere. (J) Plot profile histogram reveals the distribution of the dendritic arborizations of eight 48 h AH single motoneurons (red) and eight 96 h AH cells (yellow) within the leg neuropil. The mean centre of arbor mass for eight neurons is denoted by a coloured circle for each subtype and the standard error of each experimental condition and statistical significance between groups *** *p*<0.001. Arrowheads represent the 33^rd^ percentile for each subtype. SGV, scaled grey value. Scale bar in G applies to F. Scale bars = 20 µm. (K) Cartoon of thoracicoabdominal complex. Blue box indicates right prothoracic hemineuromere.

To establish which muscles lineage 15 motoneurons innervate, we imaged GFP labelled axons of neuroblast clones directly through the body wall and in the legs of adult flies between 2 and 4 days after eclosion. For lineage 15 motoneurons the most proximal targets are a series of body wall muscles that control the leg, including the extracoxal leg depressor (unpublished data). Axons from the remainder of lineage 15 then travel through the coxa and trochanter to the femur, where they establish connections proximally with the long tendon muscle 2, ltm2 (the pretarsal flexor), and distally with tibia reductor muscle, tirm (the accessory tibial flexor muscle) (Figure 1A and 1D). For appendicular muscle description, see Soler et al. (2004) [28]. The remaining axons pass the femoral-tibial joint and innervate all muscles in the tibia (Figure 1A and 1D). To gain insight into the central organization of this population, we visualized the projections of lineage 15 clones within the prothoracic neuromere (Figure 1H and 1K) of nervous systems counterstained with anti-neuroglian (Figure 1I). Neuroglian is a transmembrane protein that is enriched on the fasiculated primary neurites of adult-specific neurons. In the adult CNS, anti-neuroglian staining reveals a stereotyped scaffold of neurite bundles and tracts that allows us to define positions relative to the midline (Figure 1H and 1I). Neuroblast clones of lineage 15 insert their primary neurites into the anterior region of the leg neuropil (Figure 1E and 1I) and then arborize extensively within it (Figure 1E and 1H). In the anterior neuropil the bulk of the dendrites cover lateral and intermediate territories sending a few processes to the midline, whereas in the posterior there are also branches that extend to and cross the midline (Figure 1E).

To better understand the organization of lineage 15 and to establish whether its members generate a neural map, we visualized individual neurons. The induction of single-cell MARCM clones during larval life not only allowed us to reveal single cells but also enabled us to ask whether there was a sequential generation of neuronal subtypes. Focusing on the periphery first, we found that the progeny of lineage 15 innervate the proximo-distal axis of the leg in a birth-order-dependent fashion, with the first-born neurons targeting body wall muscles (unpublished data) and subsequent neurons innervating more distal targets within the leg. The neurons born following a heatshock at 48 h AH (after hatching) make synapses on ltm2 [28]. This cell appears to be a member of a subtype of neurons, of which ∼4 innervate this muscle (Figure 1A, 1B, and 1D). This observation is based on comparisons of the axonal arbors of neuroblast clones versus single neuron clones (Figure 1A and 1B), along with data from a detailed clonal analysis (Brierley et al., unpublished). The neurons generated by heatshocks at 72 h AH innervate tirm in the distal femur; this neuron is also likely to be a member of a pool of ∼4 neurons (unpublished data). The remaining neurons within lineage 15 innervate the distal most leg segment containing muscles, the tibia. The cell induced at 96 h AH is a member group of ∼3 neurons that innervates the tarsal depressor muscle of the tibia (tadm) (Figure 1A, 1C, and 1D). The observation of multiple isomorphic neurons innervating muscles in the leg is supported by a recent independent study [29] and consistent with data from larger insects [30].

Single-cell clones allowed us to visualize the dendritic projections of lineage 15 neurons with unprecedented detail. The arborizations of the first-born neurons in this lineage, labelled by heatshocks of newly hatched larvae, cover much of the leg neuropil with branches extending from the anterior medial territories (arrowhead in Figure 1E) through to the lateral edge (unpublished data). The neurons generated from a 48 h AH heatshock also span a large part of the leg neuropil running from medial territories to the lateral edge, with the majority of dendrites in an intermediate position (Figure 1F and 1H). The cells generated following a heatshock at 72 h AH span the intermediate and lateral territory but do not send branches to the midline (unpublished data). Contrasting with earlier born cells, neurons generated at 96 h AH have dendrites that are located in lateral neuropil, with some branches in the intermediate territory (Figure 1G).

We focused our studies on the neurons generated by heatshocks 48 h and 96 h AH as these are representative of two subtypes of leg motoneurons that cover clearly distinct territories within the map (Figure 1H). A more detailed description of our studies of the developmental origins and architecture of leg motoneurons will be published elsewhere (Brierley et al., in prep). We used a plot profile tool to perform quantitative analysis on the dendrites of single-cell clones. The distances along the medio-lateral (*x*) axis were first scaled from the interval 0 (midline) to 100 (lateral edge), and then weighted means were computed (using average pixel intensity as weights). These weighted means (i.e., centres of mass) were then used to compute statistical significance between profiles. We also computed the location of the mean 33^rd^ percentile pixel intensity, which was chosen as a proxy for the spread and asymmetry of the intensity profiles. Each histogram presents the accumulated data of 16 neurons (two groups of eight) and is divided into medial (M), intermediate (I), and lateral (L) neuropil territories. Analysis of 48 h AH and 96 h AH single-cell clones show how these neurons distribute their dendritic processes in a stereotyped manner across the medio-lateral axis of the leg neuropil. The mean centre of arbor mass is significantly different between the two groups (*p* = 0.00003), and the 33^rd^ percentile of the 48 h AH neurons is closer to the midline than the 96 h AH neurons (51% versus 64%).

These data revealed to us the existence of a birth-order-dependent organization of projection patterns generated by lineage 15. We have found four subtypes within this lineage based on the morphology of the dendritic arborizations and the target areas they innervate. Early-born cells innervate proximal muscle targets and elaborate dendrites from medial to lateral territories, whereas late-born cells innervate distal muscle groups within the leg and position dendrites in the lateral neuropil. Cells born between these two extremes generate dendrites that occupy intermediate positions. Taken together these data show that lineage 15 generates a myotopic map in the medio-lateral axis of the leg neuropil by birth-order-based developmental mechanisms.

### An Adult Myotopic Map Is Built by Dendritic Targeting

To gain insight into the developmental mechanisms that control the formation of myotopic maps, we looked at the way that the 48 and 96 h AH subtypes establish their dendritic arborizations. Two quite different modes of dendritic elaboration could be envisaged: dendrites could grow exuberantly initially and then eliminate branches from inappropriate territories; alternatively, they could restrict their growth to within appropriate target territories throughout their development. To establish which mode of growth these motoneurons use to generate their dendritic trees, we performed a timeline analysis through the pupal-adult transition.

**Figure 2:**
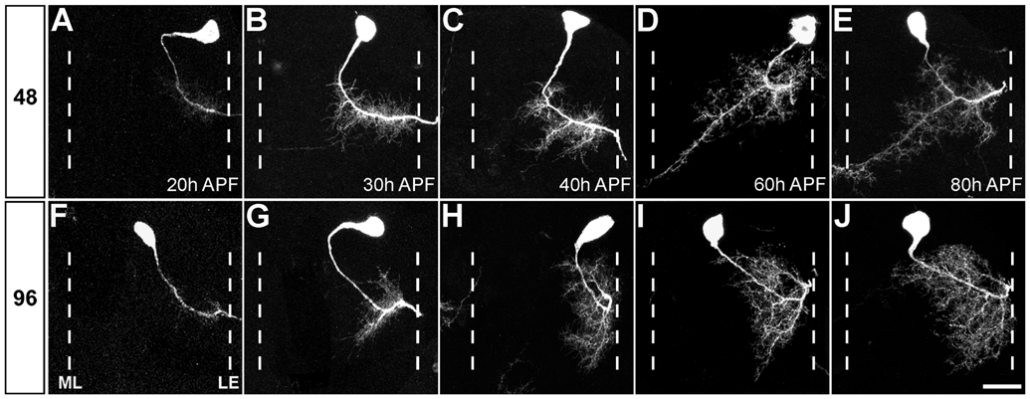
Central projections of leg motoneurons acquire their subtype-specific morphology by targeted dendritic growth. Single z-projections of leg motoneurons born following heatshocks at 48 h AH (A–E) and 96 h AH (F–J) (*n* = 4 for each). (A, F) At 20 h APF filopodia can be seen on the primary neurites of both neurons. (B, G) At 30 h APF the 48 h AH neuron generates filopodia and small branches that extend into the intermediate territory; a similar quantity of branches can be seen on the 96 h AH neuron in two growth zones at the lateral edge of the neuropil. (C, H) By 40 h APF the dendrites of both neurons have increased in size and higher order branches are beginning to be established. The 48 h AH neuron elaborates a branch on the anterior part of the primary neurite and also at two sites where the posterior branches will form. The 96 h AH cell has branches projecting to the anterior and posterior at the lateral edge of the neuropil. (D, I) By 60 h APF both neurons have increased their number of higher order branches. The major posterior branch in the 48 h AH neuron has reached the midline but not crossed it. The 96 h AH cell covers a large part of the lateral neuropil; many branches orient towards the midline but these never extend into medial territories. (E, J) At 80 h APF the morphology of both neurons are indistinguishable from those seen in the adult. Anterior is up. The relative positions of the midline (ML) and lateral edge (LE) of neuropil were determined by neuroglian staining. Scale bar = 20 µm.

At 20 h after puparium formation (APF) the axons of all leg motoneurons have left the nervous system. Both the 48 and 96 h AH neurons have generated filopodia along their primary neurites in the lateral neuropil (Figure 2A and 2F). At 30 h APF the two neurons begin to show differences in their coverage of the neuropil, with the 48 h AH neuron generating filopodia and small branches that extend into the intermediate territory, and large numbers of filopodia and small branches appear at sites where the anterior and posterior branches will form (Figure 2B). The 96 h AH cell generates a similar quantity of filopodia and small branches, but these are focused in the lateral neuropil, one oriented towards the anterior and the other to the posterior, at a site where major lateral branches will ultimately form (Figure 2G).

At 40 h APF the dendrites of both neurons have significantly increased their size and complexity and already show features that are characteristic of the adult neuron. The 48 h AH neuron elaborates a branch on the anterior length of the primary neurite, i.e., proximal to the cell body and also two growth zones at sites that will become the posterior branches, including the large one that crosses the midline (Figure 2C). At this time the 96 h AH cell has established higher order branches on both the anterior and posterior lateral branches. There are also a large number of small branches along the length of the primary neurite, many of which have filopodia extending in the direction of the midline (Figure 2H).

At 60 h APF the 48 h AH neuron has increased its number of higher order branches and the major posterior branch has reached the midline but has not crossed it. In the 96 h AH cell the dendrites covers a large part of the lateral neuropil. Higher order branches can be seen oriented towards the midline, but these never extend into medial territories (Figure 2D and 2I).

At 80 h APF, which equates to ∼80% through the pupal-adult transition, the overall morphology of both neurons is indistinguishable from those seen in adults (Figure 2E and 2J). It is possible that local refinements of the dendritic tree take place, but we did not focus on those.

These data reveal two important features of myotopic map formation in the leg neuropil: firstly, that the different subtypes of neurons within lineage 15 initiate dendritogenesis synchronously, not sequentially, regardless of their actual birth date; secondly, that both generate subtype-specific dendritic morphologies by growing into distinct territories throughout development, rather elaborating extensively and undergoing large-scale remodelling (Figure 2E and 2J). Thus, these data reveal that dendritic targeting plays a major role in generating a myotopic map in the leg neuropil.

### Molecular Mechanisms for Targeting Dendrites in the Leg Neuropil

#### a) Localization of midline signalling molecules in the developing thoracic neuropil

The dendrites of motoneurons from lineage 15 occupy specific territories along the medio-lateral axis of the leg neuropil and target their growth into these domains during adult development. We wanted to establish what molecules could control subtype-specific programs of dendritogenesis and patterning in relation to the midline. Previous work has demonstrated the role of Slit-Robo signalling in the positioning of *Drosophila* sensory axon terminals into distinct medio-lateral domains of the embryonic ventral nerve cord [24]. Slit is a diffusible repellent signal that is secreted by midline cells and Roundabout (Robo) is its evolutionary conserved transmembrane receptor, expressed on neurite growth cones [31]. Slit-Robo mediated repulsion is balanced by Netrins. The Netrins are evolutionary conserved molecules, released from midline, and act as attractants when signalling through the Frazzled(Fra)/DCC receptor, also enriched on growth cones [32]. Both signalling pathways are required in *Drosophila* embryonic motoneurons for their dendrites to cross the midline [33]. A role for these signalling systems in positioning dendrites into discrete territories to generate a neural map has not been described before.

**Figure 3:**
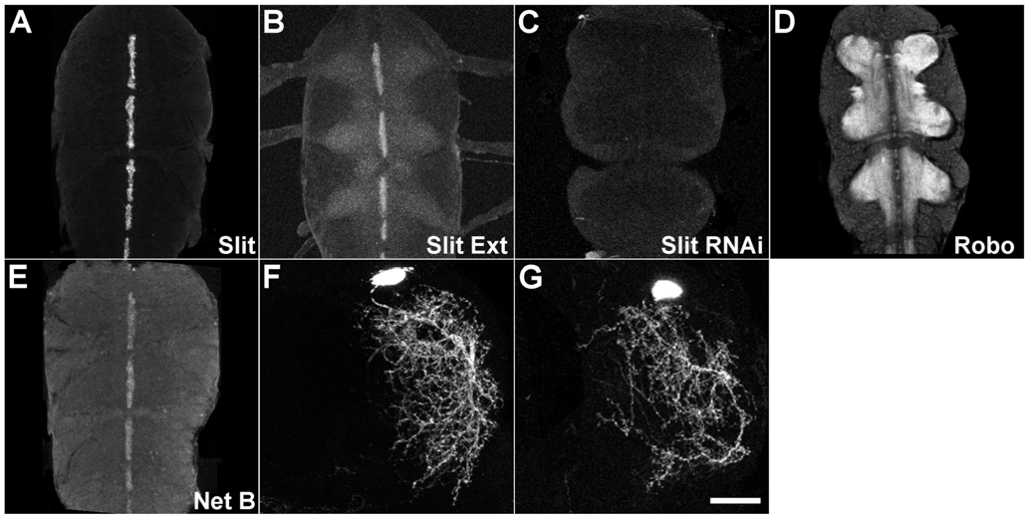
Localization of midline signalling molecules in the thoracic nervous system during the pupal-adult transition. (A) Detergent based antibody staining against Slit shows expression in midline cells at 48 h APF. (B) Detergent-free immunocytochemistry reveals robust extracellular Slit staining at the midline and within the developing neuropil at 48 h APF in the ventral nerve cord. (C) Conditionally removing Slit using RNAi. Following a knockdown of Slit, using Slit-GAL4 to drive UAS-Slit RNAi, we observed a lack of Slit staining in the developing neuropil using detergent-free immunocytochemistry. (D) Immunocytochemistry reveals Robo expression throughout all thoracic neuropils at 48 h APF. Note absence of staining at the midline. (E) Anti-myc staining reveals the localization of NetrinB in midline glia at 48 h APF, in a NetrinB-myc allele. (F) Ectopic expression of Robo::myc fusion protein in single-cell clone reveals that Robo can be localized in dendrites of leg motoneurons. (G) Ectopic expression of Fra::myc fusion protein in a single-cell motoneuron clone. Anterior is up. Scale bar applies to F and G = 20 µm.

We first established that these signalling molecules are indeed present in the leg neuropil when leg motoneuron dendritogenesis takes place. Antibodies against Slit and Robo were used to determine the localization of these proteins during the pupal to adult transition. Robust expression of Slit was detected in the midline cells of each thoracic neuromere using detergent-based immunohistochemistry (Figure 3A). We also visualized extracellular Slit using a detergent-free immunohistochemical method, where only secreted Slit should be revealed. In this case we not only found staining at the midline but also observed low levels of Slit throughout the neuropil (Figure 3B). To confirm that the staining in the neuropil was genuine, we reduced the levels of Slit in the developing CNS by expressing RNAi against Slit from the beginning of the third instar. We then stained using the detergent-free immunohistochemical method and found a robust loss of immunoreactivity at the midline and in the neuropil, indicating that the staining observed in the wild-type condition was bona fide extracellular Slit protein (Figure 3C). Staining with an antibody against Robo revealed the receptor to be present throughout the whole leg neuropil, with an absence of staining at the midline (Figure 3D). To visualize Netrin we used a myc-tagged allele of NetrinB [34] and saw staining in midline cells (Figure 3E). Although the Robo antibody data clearly revealed robust staining in the neuropil, we wanted to establish whether Robo could be localized to the dendrites of leg motoneurons. To do this we expressed a myc-tagged version of Robo in a single-cell clone induced at 96 h AH. Anti-myc staining revealed the localization of the Robo fusion protein in the dendrites of this cell (Figure 3F). Similarly, when we used a myc-tagged version of Frazzled, we also found it in dendrites (Figure 3G). Taken together these data reveal that a number of the components required for midline signalling are present in the leg neuropil at the time when motoneurons are undergoing dendritogenesis and could potentially play a role in targeting dendrites into the appropriate territories during map formation.

#### b) The role of Slit-Robo signalling in targeting motoneuron dendrites in the leg neuropil

To establish if Slit-Robo signalling is required for targeting leg motoneuron dendrites to distinct territories within the medio-lateral axis, we performed clonal analysis using MARCM and generated single-cell clones homozygous with either *robo^GA2875^* or *robo^ZI772^*, null alleles of the slit receptor Robo [31]. This powerful in vivo method allowed us to study the behaviour of individual mutant cells in a wild-type background. Our experiments revealed that both alleles show the same phenotype (unpublished data), and for the rest of the study we made clones with *robo^GA2875^*. We used the plot profile tool to compare Robo loss of function (RoboLOF) clones with eight single-cell wild-type clones.

**Figure 4:**
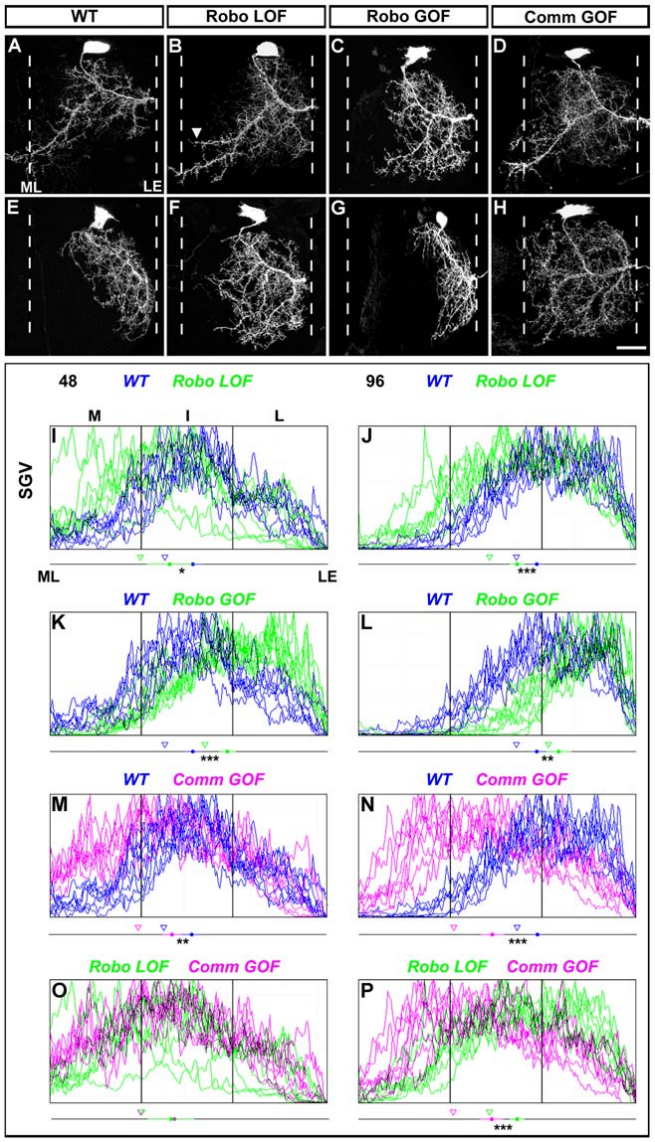
Slit-Robo signalling mediates dendritic targeting of motoneurons within the medio-lateral axis of the leg neuropil. (A) Motoneurons born following heatshocks at 48 h AH elaborate dendrites across all three neuropil territories and send a large posterior branch toward the midline. (B) Robo null clone generated at 48 h AH generates ectopic dendritic projections near the midline at the posterior region of the neuropil (arrowhead). (C) Robo gain of function clones generated at 48 h AH lack the midline crossing event in the posterior region of the neuropil. (D) Misexpression of Comm in motoneurons generated at 48 h AH clones results in inappropriate growth of dendrites towards medial regions of the neuropil. (E) Motoneurons born following a heatshock at 96 h AH elaborate dendrites in intermediate and lateral territories within the leg neuropil. (F) Robo null clones generated at 96 h AH show medial shifts in the distribution of their dendrites. (G) Misexpression of Robo in 96 h AH clones results in dendrites being shifted away from the midline into very lateral neuropil territories. (H) Misexpression of Comm in the 96 h AH clones results in dendrites that span the medio-lateral axis of the neuropil. (I–P) Plot profile graphs to reveal the distribution of dendrites along the medio-lateral axis within the leg neuropil (*n* = 8 for each genotype). The mean centre of arbor mass (circle) for the dendritic arbors and the standard error of each experimental condition and the statistical significance between groups are also shown. Triangles denote the 33^rd^ percentile for each group. Scale bar = 20 µm. * *p*<0.05, ** *p*<0.01, and *** *p*<0.001.

In the 48 h RoboLOF motoneurons, the posterior dendritic branch crosses the midline at the same site as it does in wild-type clones (Figure 4A and 4B), but the loss of Robo also leads to ectopic branches that extend toward the midline at more anterior positions within the neuropil (arrowhead, Figure 4B). The plot profiles reveal that more of the dendritic arborization is found in the medial territory than controls (Figure 4I). The mean centre of mass in these RoboLOF clones was shifted toward the midline compared to controls and a *t* test between the two groups shows they are significantly different (*p* = 0.029). The mean 33^rd^ percentile of arborization was shifted towards the midline in the RoboLOF group (33% versus 41%) (Figure 4I).

In the 96 h RoboLOF clones there was a shift in the distribution of dendrites towards the midline (Figure 4F and 4J) compared to wild-type, where the majority of the dendrites elaborate in the lateral region of the neuropil (Figure 4E and 4J). The mean centre of mass of the RoboLOF 96 h group shifts towards the midline compared to controls and the two groups are significantly different (*p* = 0.00008) (Figure 4J). The mean 33^rd^ percentile of the arborization in the RoboLOF group also shifts towards the midline (47% versus 57%) (Figure 4J). Throughout these studies we saw no evidence for a disruption to the axonal projections of lineage 15 RoboLOF clones in the periphery.

To determine if an increase in Robo signalling is sufficient to change the distribution of the dendrites, we performed gain of function experiments where we overexpressed wild-type Robo in individual cells (RoboGOF). In the 48 h AH neuron Robo overexpression resulted in a general shift of the arborization away from the midline with an increase in the posterior lateral regions of the neuropil (Figure 4C and 4K). Plot profile data reveal a shift in the mean centre of mass away from the midline and that these groups are significantly different from each other (*p* = 0.00003). The mean 33^rd^ percentile of arborization shifts away from the midline (56% versus 41%; Figure 4K). In the 96 h neurons, overexpressing Robo was also sufficient to shift dendrites away from the midline (Figure 4G). Plot profile data show statistically significant (*p* = 0.001) shifts in the mean centre of mass and the mean 33^rd^ percentile of arborization (68% versus 57%) away from the midline (Figure 4L).

We next wanted to address whether Slit-Robo signalling in these cells could be regulated by the negative regulator of Robo, Commisureless (Comm) [35]. To do this we ectopically expressed Comm in both the 48 and 96 h AH subtypes (CommGOF). Within the embryonic nervous system of *Drosophila*, Comm plays a key role in down regulating Robo signalling in contralaterally projecting neurons, allowing their growth cones to cross the midline [35]. We found that Comm overexpression resulted in the dendrites of 48 h AH neurons shifting towards the midline (Figure 4D and 4M). The mean centre of mass of the 48 h AH CommGOF neurons moved towards the midline and was significantly different to the control group (*p* = 0.003). The mean 33^rd^ percentile also shifts towards the midline (32% versus 41%; Figure 4M).

In 96 h AH CommGOF single-cell clones we found the dendrites entered medial territories within the neuropil with several branches terminating on the midline. The mean centre of mass was shifted to the midline and was significantly different to the control group (p = 0.000001; Figure 4N). The mean 33^rd^ percentile also shows a shift towards the midline (34% versus 57%; Figure 4N). Using plot profile we compared CommGOF and RoboLOF clones. The mean centre of mass in the 48 h AH CommGOF and RoboLOF appear identical and the two groups are clearly not significantly different (*p* = 0.7). The mean 33^rd^ percentiles for both groups were very similar (32% versus 33%) (Figure 4O). A comparison of CommGOF and RoboLOF 96 h AH neurons shows that the mean centre of mass of the CommGOF is nearer the midline than RoboLOF and that the two groups are significantly different (*p* = 0.0002). The mean 33^rd^ percentile is different, with CommGOF being closer to the midline (33% versus 47%; Figure 4P).

In summary these data reveal that Slit-Robo signalling is required in both the 48 and 96 h AH motoneurons for the generation of the appropriate medio-lateral position of their dendritic arborizations. We find that increasing the levels of Robo is sufficient to cause a shift in dendrite distribution and that Comm overexpression is capable of down regulating Robo receptors in the these motoneurons. Interestingly, Comm overexpression generates more severe phenotypes than the loss of Robo receptor alone in neurons born at 96 h AH (Figure 4H).

#### c) The role of Netrin-Fra signalling in targeting motoneuron dendrites

**Figure 5:**
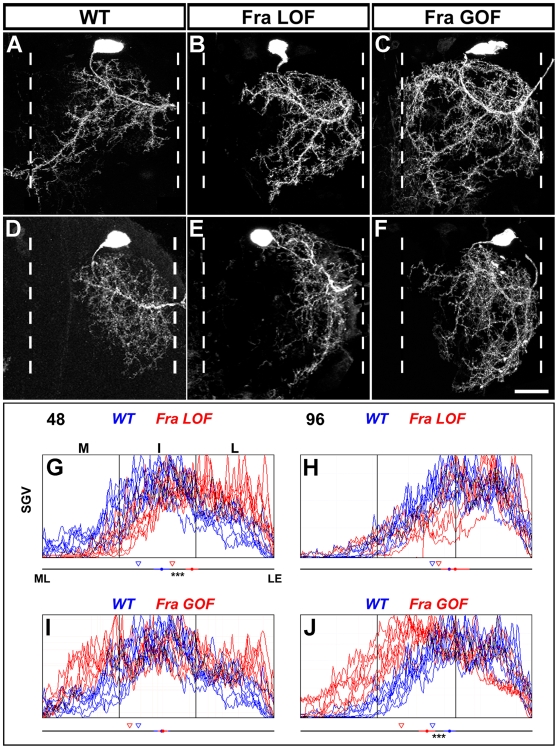
Netrin-Fra signalling mediates dendritic targeting of motoneurons within the medio-lateral axis of leg neuropil. (A) Motoneurons born following heatshocks at 48 h AH elaborate dendrites across all three neuropil territories and send a large posterior branch toward the midline. (B) In Fra null single-cell clones generated at 48 h AH the posterior dendritic branch fails to cross the midline and show an increase in the amount of arborization in the lateral regions of the neuropil. (C) Misexpression of Fra in single 48 h AH results in a redistribution of dendrites to both lateral and medial territories. (D) Motoneurons born at 96 h AH elaborate dendrites that target lateral territories within the leg neuropil. (E) In Fra null single-cell clones generated at 96 h AH the dendrites show no obvious change in dendrite position along the medio-lateral axis. (F) Misexpression of Fra in 96 h AH clones results in dendrites shifting towards medial territories. (G–J) Plot profile graphs to reveal the dendrite distribution along the medio-lateral axis within the leg neuropil (*n* = 8 for each genotype). The mean centre of mass (circle) for the dendritic arborizations for each genotype along the medio-lateral axis is shown on the line underneath the histogram. The standard error of each experimental condition and the statistical significance between groups is also shown. Triangles denote the 33^rd^ percentile for each group. Scale bar = 20 µm.

To investigate whether Netrin-Fra signalling is required for the appropriate targeting of motoneuron dendrites in the leg neuropil, we generated single-cell clones homozygous for either *fra^3^* and *fra^4^*, null alleles of Frazzled, a Netrin receptor. Our preliminary experiments revealed that both alleles show the same phenotype (unpublished data) and for the rest of the study we made clones with *fra^3^*. In the 48 h AH Frazzled loss of function clones (FraLOF), the posterior dendritic branch fails to approach and cross the midline. These FraLOF neurons also showed an increase in dendritic arborization in the lateral regions of the neuropil (Figure 5A and 5B). The plot profiles reveal that the mean centre of mass of these neurons is shifted away from the midline and both groups are significantly different from each other (*p* = 0.00002; Figure 5G). The mean 33^rd^ percentile of arborization was also shifted away from the midline in the FraLOF group (56% versus 41%; Figure 5G). Interestingly, the 96 h FraLOF neurons do not show a major change in position of their dendrites (Figure 5D and Figure 5E). The mean centre of mass and mean 33^rd^ percentile reveals only a small shift away from the midline, and the experimental and control group are not significantly different from each other (*p* = 0.33) (Figure 5H).

We next asked whether increasing Fra was sufficient to shift the position of the dendrites along the medio-lateral axis. To do this we generated single-cell clones expressing wild-type Frazzled (FraGOF). This treatment had a major effect on the dendritic arborization of both 48 and 96 h AH neurons. The dendrites of the 48 h AH cells elaborate branches in anterior medial territories, and the posterior branch also fails to leave and cross the midline. Interestingly, this treatment also results in a greater number of branches found in the posterior lateral territory (Figure 5C). The mean centre of arbor mass in this case was the same in experimental and control groups, and statistically there was no significant difference (Figure 5I). This reveals the limitations of using centre of arbor mass analysis alone as it does not extract the difference between these two groups. The dendrites of 96 h AH FraGOF cells are shifted into intermediate and medial territories with the mean centre of mass moving to the midline (Figure 5F). The two groups were significantly different (*p* = 0.00003; Figure 5J). The 33^rd^ percentile shows a shift to the midline.

In summary, these data show that Netrin-Fra signalling is required for the normal targeting of the dendrites of the 48 h AH subtype into its appropriate medio-lateral position but does not play a major role in establishing the territory of the cells born at 96 h AH.

#### d) The role of the guidance molecules Slit and Netrin in targeting motoneuron dendrites

Our functional analysis of Slit-Robo and Netrin-Fra signalling has thus far focussed on manipulating the levels of the receptors Robo and Fra in single cells. We next wanted to establish whether similar disruptions would occur if we removed the ligands Slit and Netrin.

**Figure 6:**
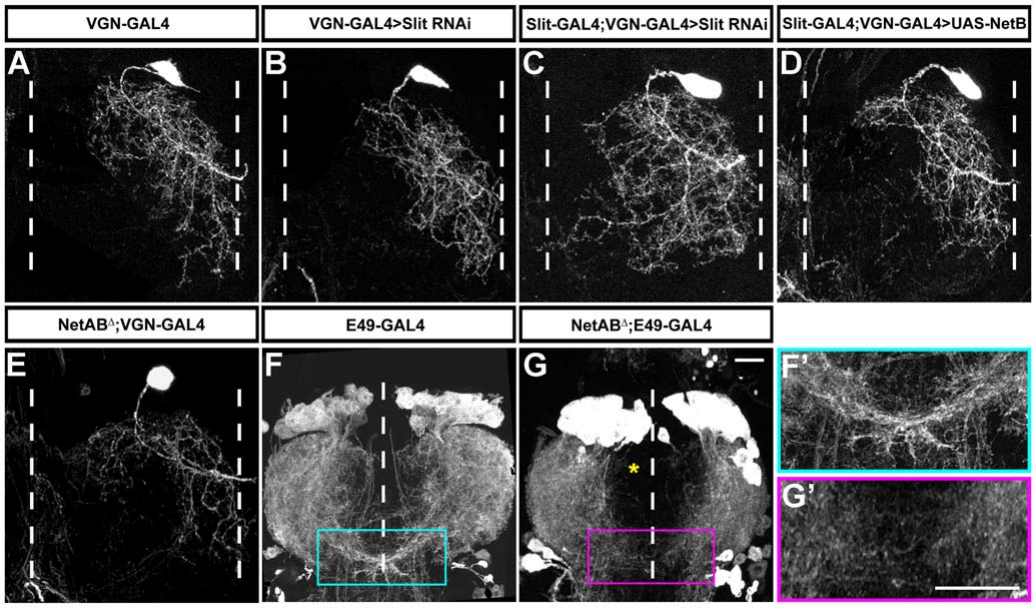
Slit and Netrin are required for motoneuron dendritic targeting. (A) VGN9281-GAL4 expressing CD8::GFP. The motoneuron labelled by VGN9281-GAL4 targets dendrites to lateral territories of the leg neuropil. (B) VGN9281-GAL4 expressing CD8::GFP and Slit-RNAi. Expressing Slit-RNAi in the motoneuron has no effect on medio-lateral distribution of its dendrites. (C) Slit-GAL4 and VGN9281-GAL4 expressing CD8::GFP and Slit-RNAi. Reducing Slit protein in the midline cells results in dendrites of the *VGN*9281-GAL4 motoneuron being shifted into intermediate and medial territories. (D) Expressing CD8::GFP and NetrinB under the control of Slit-GAL4 and VGN9281-GAL4 does not change the medio-lateral position of the dendrites of the motoneuron. (E) VGN9281-GAL4 expressing CD8::GFP in NetABΔ background. The dendrites of the VGN9281-GAL4 motoneuron do not change position in a NetABΔ background. (F) E49-GAL4 expressing CD8::GFP reveals the bulk of the motoneurons of the prothoracic neuromere. The dendrites of the motoneurons that innervate the pretarsal flexor muscles approach and cross the midline. Boxed area detail shown in F'. (G) E49-GAL4 expressing CD8::GFP in a NetABΔ background reveals the dendrites of the pretarsal flexor muscle motoneurons fail to approach and cross the midline. Asterisk denotes anterior part of neuropil where dendrites fail to approach the midline. Boxed area detail shown in G'. (F') Detail from F. (G') Detail from G. Scale bars = 20 µm. Scale bar in E applies for A–E. Scale bar in G applies to F. Scale bar in G' applies to F'.

Slit nulls are homozygous lethal and this prevented us from performing analysis on adult motoneuron dendrites. To overcome this we have established a method to conditionally knock down Slit using RNAi during adult development. To do this we expressed Slit RNAi in the midline cells under the control of the Slit-GAL4 driver. As described above our antibody staining revealed that Slit is not detectable by 48 h APF in such animals (Figure 3C). To visualize a late-born neuron we used VGN9281-GAL4, which contains a fragment of the *Drosophila* VGlut *cis*-regulatory region. As well as labelling a late-born cell of the 96 h AH subtype (Figure 6A), it also expresses GAL4 in a small number of interneurons. Using this tool we were able to knock down Slit protein (Slit-GAL4>UAS Slit RNAi) and simultaneously visualize the dendrites of the motoneuron labelled by VGN9281-GAL4. We found that when we removed Slit, the dendrites of the motoneuron shifted into intermediate and medial neuropil territories with several branches projecting to and terminating at the midline (Figure 6C). As a control we expressed UAS-Slit RNAi in VGN9281-GAL4 alone and found the distribution of the dendrites the same as wild-type (Figure 6B).

We next wanted to establish whether changing the levels of Netrin would also affect the distribution of dendrites. Firstly, we expressed NetrinB in the midline cells and found the dendrites of the motoneuron labelled by VGN9281-GAL4 had a wild-type distribution (Figure 6D). We were able to remove both NetrinA and NetrinB using the mutant NetABΔ [34] as a small number of NetABΔ males eclose (Alex Mauss pers comm.). We found that the loss of both Netrins did not affect the placement of dendrites within the medio-lateral axis compared to wild-type (Figure 6A and 6E). This result appears consistent with the earlier observation of the loss of the Netrin receptor Fra on the late-born cells (Figure 5E and 5H). We next wanted to establish whether the dendrites of cells that project to the midline were disrupted in the NetABΔ mutant. To do this we used E49-GAL4 [36], which appears to label most motoneurons in the prothoracic neuromere (Figure 6F and 6F'), including the subtype that innervates the pretarsal muscles, whose dendrites project to and across the midline (Figure 6F'). We found that these distinctive midline-projecting dendrites were missing in the NetABΔ male escapers, as well as more medially projecting dendrites in the anterior regions of the neuropil (Figure 6G and 6G'). It would therefore appear that Slit is important for the correct targeting of the dendrites of late-born motoneurons within lineage 15, whereas the levels of Netrin are important for targeting medially projecting dendrites.

#### e) The role of Slit-Robo signaling in dendritic growth and complexity

When we removed Robo or ectopically expressed Comm, the arborizations of leg motoneurons appeared larger and more complex (Figure 4F and, 4H). We wanted to know whether this increase in size could be the reason why we observed a shift in dendrite distribution along the medio-lateral axis, i.e., that an increase in growth (i.e., dendrite mass) causes dendrites to “spill over” into medial territories. This is quite a different scenario to one where dendrites remain the same mass but occupy a different spatial coordinates due to a change of guidance cue signalling.

**Figure 7:**
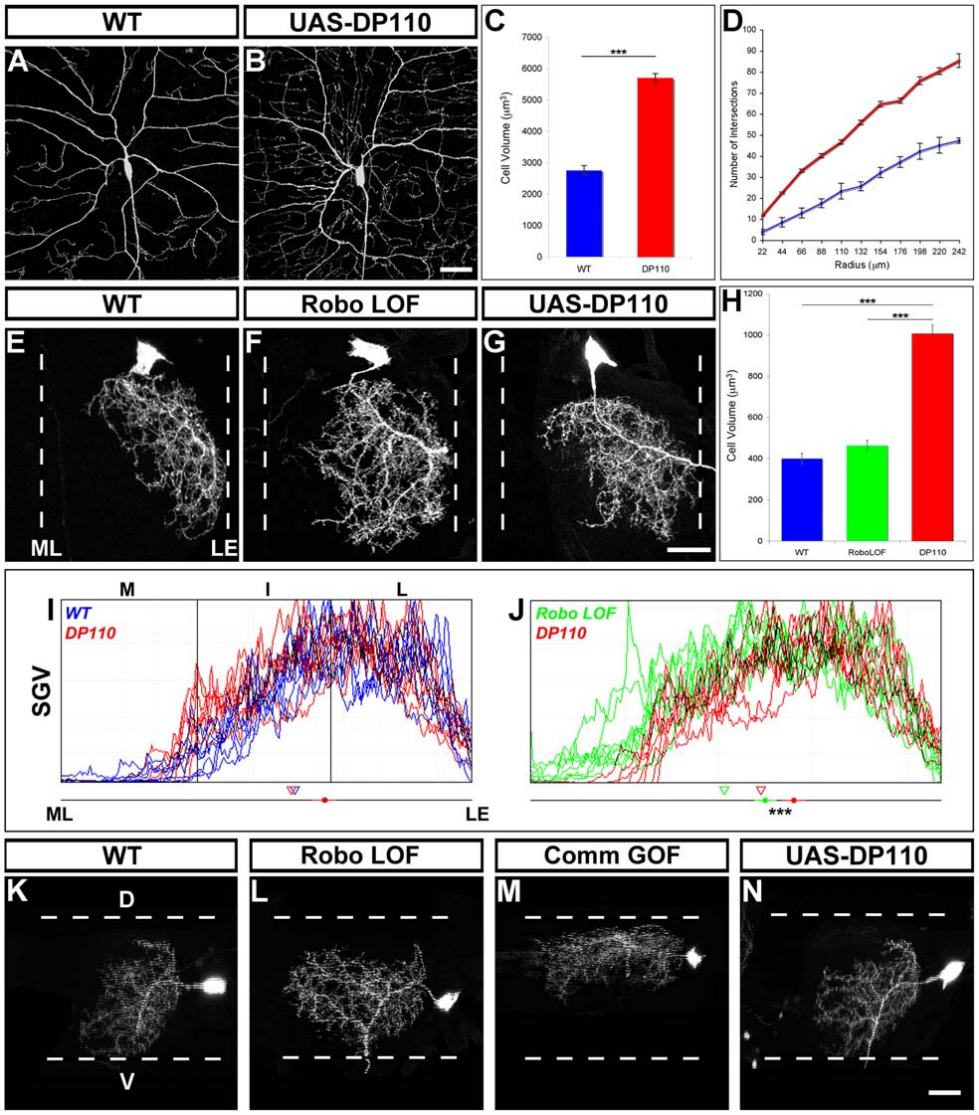
The positioning of leg motoneuron dendrites occurs independently of changes in dendritic mass. (A) Detail of the proximal dendrites of the sensory neuron ddaC. WT, wild-type. (B) Detail of the proximal dendrites of the sensory neuron ddaC expressing Dp110 reveals an increase in branch complexity. (C) Analysis of cell body size in wild-type (blue) and DP110 expressing (red) ddaC neurons. (D) Sholl analysis of wild-type (blue) and DP110 expressing (red) ddaC neurons. (E) Motoneurons born at 96 h AH generate dendrites that target lateral neuropil territories. (F) Robo null (RoboLOF) clones generated at 96 h AH show medial shifts in the distribution of their dendritic fields. (G) Ecotopic expression of UAS-DP110 in 96 h AH clones results in dendritic arborizations that elaborate mainly in lateral territories of the neuropil with a few ectopic medial branches present in anterior and posterior neuropil. (H) Analysis of cell body size in wild-type (WT), RoboLOF, and UAS-DP110 96 h AH clones. (I–J) Plot profile graphs to reveal the distribution of dendrites along the medio-lateral axis within the leg neuropil (*n* = 8 for each genotype). The mean centre of mass (circle) for the dendrites of each genotype. The standard error of each experimental condition and the statistical significance between groups can also be seen. Triangles denote the 33^rd^ percentile for each group. (K) Dorsoventral projection of a wild-type dendritic arborization of a motoneuron born at 96 h AH. (L) Dorsoventral projection of a Robo LOF 96 h AH motoneuron. (M) Dorsoventral projection of a CommGOF 96 h AH motoneuron. (N) Dorsoventral projection of a DP110 GOF 96 h AH motoneuron. (K–N) Dorsal is up. Anterior to right. Scale bars = 20 µm.

To test this idea we next set out to establish if an induced increase in dendrite mass alone would result in dendrites “spilling over” into medial territories. We predicted that the cell autonomous activation of the insulin signalling pathway would allow us to change the global size of the cell whilst leaving other features of the environment the same. To do this we expressed the active subunit of PI3-Kinase, Dp110, which is sufficient to enlarge many different cell types [37]. To first determine how Dp110 changes neuronal morphology, we expressed it in the class IV dendritic arborizing sensory neuron ddaC, as the dendrites of these cells have a nearly two-dimensional organization, making them very amenable to morphometric analysis [38]. Using Volocity Acquisition software (version 4.2, Improvision), we imported raw data stacks and reconstructed 3D projections of the cell bodies of ddaC neurons from both control and experimental groups (Figure 7A and 7B). We then used the lasso tool to capture the cell body and subsequently measure the volume and found that control ddaC neurons had a mean soma volume of 2760.9±149.9 µm^3^ whereas the mean soma volume in the Dp110 group was 5702.2±142.3 µm^3^ (Figure 7C). To establish how the dendrites of the two groups were different, we performed Sholl analysis [39] to give a quantitative measure of dendrite complexity throughout the tree. The number of intersections between dendritic processes and Sholl-rings were counted in Photoshop using a template of 12 concentric circles (each 22 µm apart) centred on the cell body. We found the Dp110 expressing neurons distributed their branches in much the same manner as wild-type neurons, but had a two-fold increase in branch number (Figure 7A, 7B, and 7D). Thus Dp110 overexpression results in a two-fold increase in soma size that was mirrored with a two-fold increase in dendritic branch number.

We used this approach to enlarge single 96 h AH motoneurons from lineage 15. The mean soma volume of this motoneuron increased from 399.3±25.9 µm^3^ to 1005.2±42.9 µm^3^ (∼two-fold increase) when Dp110 was expressed (Figure 7E, 7G, and 7H). This increase of soma size also appears to be mirrored by an increase in the complexity of branching (Figure 7G). We performed quantitative analysis using the plot profile tool to establish if the increase in dendrite mass had changed the medio-lateral distribution of dendrites. This comparison revealed a few Dp110 expressing neurons increased the quantity of dendrites in the intermediate territory, but overall there is no major shift to the midline and no statistical difference in the mean centre of arbor mass between groups (*p* = 0.93; Figure 7I). The difference between the RoboLOF and the Dp110 group is marked, with plot profiles of the RoboLOF clearly shifted to the midline compared to the Dp110 group and the mean centre of arbor mass has also shifted with the two groups being significantly different from each other (*p* = 0.0001; Figure 7J). An analysis of the wild-type versus RoboLOF soma size also shows there to be no statistical difference between the two groups (Figure 7H). In summary these data suggest that increasing the mass of the dendrites alone did not result in significant medial shifts of motoneuron dendrites.

We wondered whether the apparent increase in dendrite complexity seen in the RoboLOF and CommGOF 96 h AH clones (Figure 4F and 4H) could be due to a redistribution of the dendrites in another axis. To address this we projected our datasets through 90 degrees and looked at the distribution of dendrites in a dorso-ventral plane. We found that wild-type clones cover a large area of the dorso-ventral axis of the leg neuropil with a bias towards the anterior region (Figure 7K). The RoboLOF clones showed a shift towards the posterior neuropil and a slight loss in the ventral neuropil (Figure 7L). The CommGOF clones (Figure 7M) showed a redistribution of dendrites into posterior and very dorsal neuropil, whilst arbor was entirely lost from ventral domains (also seen in FraGOF 96 h AH neurons, unpublished data). In contrast the Dp110 expressing neurons were more complex but covered much the same territory as wild-type clones (Figure 7N). In summary changes in the distribution of the dendrites of 96 h AH neurons along the medio-lateral axis correlate with changes in distribution along the dorso-ventral axis of the neuropil.

## Discussion

Neural maps are emergent, highly ordered structures that are essential for organizing and presenting synaptic information [40],[41]. The architecture of dendrites and the role they play in establishing connectivity within maps has been somewhat overlooked [2],[7]. Classic cell-labelling studies in the moth *Manduca sexta* revealed that the dendrites of motoneurons are topographically organized to reflect their site of innervation in the bodywall [42]. More recent work by Landgraf and colleagues has demonstrated unequivocally that motoneurons in *Drosophila* embryos generate a detailed dendritic (myotopic) map of body wall muscles within the CNS [18]. Alongside these data, studies on the architecture of the spinal cord also suggest that similar design principles may play a role in organizing information in vertebrate motor systems [21],[43],[44]. How such dendritic maps are built is still largely unknown.

Here we describe for the first time the role dendritic targeting plays in constructing a myotopic map and the molecular mechanisms that control it.

### Birth-Order Dependent Projections of a Lineage Generate a Myotopic Map

The majority of leg motoneurons in a fly are born postembryonically and most of those are derived from a single neuroblast lineage, termed lineage 15 [23, Brierley et al., in prep]. Perhaps the most striking feature of this lineage is its birth-order-based pattern of innervation along the proximo-distal axis of the leg. Using mosaic analysis we observed the sequential production of four neuronal subtypes during larval life, each elaborating stereotyped axonal and dendritic projections in the adult. The axon of the first-born neuron innervates a muscle in the body wall and subsequent neurons innervate more distal targets in the leg. This organization has also been recently reported by Baek and Mann (2009) [29].

This birth-order-based peripheral pattern of lineage 15 is mirrored in the CNS, where dendrites generate a stereotyped anatomical organization. Dendrites of early-born cells span medial to lateral territories, whereas late-born cells elaborate dendrites in the lateral neuropil and cells born between these times occupy intermediate territories. The sequential production of neuronal subtypes by neural precursor cells is a common mechanism for generating a diversity of circuit components [45]. A similar birth-order-based specification of axonal and dendritic projection patterns has previously been described for projection neurons in the fly's olfactory system [46],[47].

Our data reveal the existence of a myotopic map in the adult fly and supports the proposition that dendritic maps are a common organizing principle of all motor systems [18]. Mauss et al. (a companion paper) also reveal a map in the embryonic CNS of *Drosophila*, where the dendrites of motoneurons are organized along the medio-lateral axis of the neuropil representing dorsoventral patterns of innervation in the body wall muscles.

### A Neural Map Built by Dendritic Targeting

How are dendritic maps built? The myotopic map we see in the leg neuropil could be generated by two distinctly different mechanisms. Neurons could elaborate their dendrites profusely across a wide field and then remove branches from inappropriate regions or, alternatively, they could target the growth of dendrites into a distinct region of neuropil throughout development. Both mechanisms can generate cell-type-specific projection patterns as seen in the vertebrate retina [13],[15],[48]. To reveal which mechanism is deployed in the leg motor system of *Drosophila*, we imaged single-cell clones of motoneuron subtypes generated by heatshocks at 48 and 96 h AH, as their final dendritic arborizations cover clearly distinct territories within the map. The dendrites of both elaborate branches only in territories where the mature arborizations eventually reside, which strongly supports the notion that this myotopic map is generated by targeting and not large-scale branch elimination. Importantly, this developmental timeline also revealed that the motoneurons elaborate their dendrites synchronously, regardless of the birth date of the cell. This observation suggests that a “space-filling/occupancy based” model, where later-born neurons are excluded from medial territories by competitive interactions is unlikely. Similarly, heterochronic mechanisms where different members of the lineage experience different signalling landscapes due to differences in the timing of outgrowth are not likely either. With synchronous outgrowth dendrites experience the same set of extracellular signals, suggesting that the intrinsic properties of cells, defined by their birth order, may be more important for the generation of subtype-specific projections. Such intrinsic properties could include cell-cell recognition systems such as adhesion molecules, e.g., Dscams [49] or classical guidance receptors [5],[7], that could interpret extracellular signals. In the *Drosophila* embryo motoneurons also use dendritic targeting to generate a myotopic map [see Mauss et al.].

### Midline Signalling Molecules Control Dendrite Targeting and Myotopic Map Formation

It is emerging that dendrites are guided by the same molecules that control axon pathfinding [7],[12],[33]. The medio-lateral organization of leg motoneuron dendrites within the leg neuropil prompted us to ask whether the midline signalling molecules Slit and Netrin and their respective receptors Roundabout and Frazzled could be involved in targeting growth to specific territories.

Using mosaic analysis we found that both the 48 and 96 h AH motoneuron subtypes require Robo to generate their appropriate shape and position within the medio-lateral axis. When we removed Robo from the 48 h AH subtype the mean centre of arbor mass was shifted toward the midline. The dendrites of 96 h AH neurons showed a shift in distribution in the absence of Robo but still failed to reach the midline, suggesting that only part of this cell's targeting is due to repulsive cues mediated by the Robo receptor. We predicted that if Robo levels played an instructive role in dendrite targeting we would be able to shift dendrites laterally by cell autonomously increasing Robo. We found this to be the case in both subtypes. Taken together these data suggest that differences in the level of Robo signalling may provide a mechanism by which Slit could be differentially interpreted to allow subtype-specific targeting along the medio-lateral axis.

The Robo receptor is part of a larger family of receptors that includes Robo2 and Robo3 [50],[51]. This family of receptors have been found to be important for targeting axons to the appropriate longitudinal pathway in the embryonic CNS [50],[51]. Comm plays a key role in allowing contralaterally projecting neurons to cross the midline [35], and its ectopic expression (CommGOF) is known to robustly knock down Robo [31] and Robo2 and 3 [52]. We cell autonomously expressed Comm in both lineage 15 subtypes and found shifts to the midline in both 48 and 96 h AH neurons. For the 48 h AH neurons, Robo LOF data and CommGOF data are comparable, suggesting that Robo alone plays a major role in the positioning dendrites of these cells. In contrast, in the 96 h AH subtype we found RoboLOF and CommGOF effects to be significantly different, suggesting that the 96 h AH subtype may not only use the Robo receptor but additional Robos as well. Our knockdown of Slit also supports this idea, as we occasionally find the branches of late-born neurons reaching the midline (Figure 6C), something we never see in RoboLOF clones. Thus, one way of establishing differences in the medio-lateral position could be through a dendritic “Robo code” where early-born cells express Robo and late-born cell express multiple Robo receptors.

With Netrin being expressed in the midline cells during the pupal-adult transition we wondered whether attractive Netrin-Fra signalling could also contribute to positioning dendrites in the leg neuropil. When Fra was removed from the 48 h AH subtype we found that the arborization was shifted laterally, whereas removing it from the 96 h AH subtype had little effect, neither did the removal of Netrin A and B from the midline (Figure 6E), suggesting that Netrin-Fra signalling may not play a role in dendritic targeting in the later-born cell. It may be that Fra is expressed in early-born cells within the lineage and then down-regulated, although we cannot exclude that Netrin-Fra signalling masked by the repulsion from Slit-Robo signalling. These data are consistent with Fra being a major player in targeting the dendrites of the 48 h AH cell. The fact that both Fra and Robo are required for normal morphogenesis of 48 h AH neurons raises the possibility that members of lineage 15 could use a “push–pull” mechanism for positioning their dendrites, where the blend of receptors within a cell dictates the territory within the map that they will innervate.

How could such subtype-specific blends of receptors be established? A number of studies have revealed that spatial codes of transcription factors are important for specifying the identity of motoneuron populations [53]–[55]. Within lineage 15 it is possible that temporal, rather than spatial, transcription factor codes are important for regulating the blend of guidance receptors. A number of molecules have been identified that control the sequential generation of cell types within neuroblast lineages [45],[56]. Chief amongst these are a series of transcription factors that include Hunchback, Krüppel, Pdm, Castor and Seven-up. These temporal transcription factors are transiently expressed within neuroblasts and endow daughter neurons with distinct “temporal identities”. Castor and Seven-up are known to schedule transitions in postembryonic lineages, regulating the neuronal expression of BTB-POZ transcription factors Chinmo and Broad [57],[58]. It is possible that the temporal transcription factors Broad and Chinmo could control the subtype-specific expression of different Robo receptors or the Netrin receptor Frazzled in leg motoneurons. There is a precedent for this in the *Drosophila* embryo, where motoneuron axon guidance decisions to distal (dorsal) versus proximal (ventral) targets are orchestrated by Even-Skipped, a homeobox transcription factor [59], which in turn controls the expression of distinct Netrin receptor combinations [60].

Studies focusing on the growth of olfactory projection neuron dendrites in *Drosophila* reveal that they elaborate a glomerular protomap prior to the arrival of olfactory receptor neurons [14] suggesting that target/partner-derived factors may not be necessary for establishing coarse patterning of synaptic specificity. The global nature of the signals we describe here and their origin in a third-party tissue is a fundamentally different situation to that where target-derived factors instruct partner cells, such as presynaptic amacrine cells signalling to retinal ganglion cell dendrites in the zebrafish retina [15]. Furthermore, although we show that Slit and Netrin control the positioning of dendrites across the medio-lateral axis of the CNS in this study, it may be that other similar guidance signals are important for patterning dendrites in other axes [61]. There is a striking conservation of the molecular mechanisms that build myotopic maps in the embryo and pupae [see article by Mauss et al.]. Understanding the similarities and differences between these myotopic maps, from an anatomical, developmental, and functional perspective, may give us insight into the evolution of motor systems and neural networks in general.

### The Role of Slit-Robo Signalling in Dendrite Growth and Complexity

In our study we found that individual leg motoneurons that lacked Robo signalling appeared to have more complex dendritic arborizations. Our working hypothesis, that dendrites invaded medial territories because of a failure of Slit-Robo guidance function, did not take into account the possibility that cells may generate more dendrites due to a change in a cell-intrinsic growth program. Thus the changes we see in dendrite distribution relative to the midline could formally be a result of “spill-over” from that increase in cell size/mass. To determine whether this was the case we generated larger cells by activating the insulin pathway in single motoneurons. We found the dendrites of these “large cells” remained within their normal neuropil territory, supporting the idea that the removal of Robo-Slit signalling results in a disruption in guidance, not growth. These data underline the fundamental importance of midline signals in controlling the spatial coordinates that these motoneuron dendrites occupy, i.e., that a neuron twice the size/mass of a wild-type cell is still marshalled into the same volume of neuropil.

When we reconstructed our image stacks to look at the distribution of the dendrites in the dorso-ventral axis, we found that the apparent increase in size was in fact a redistribution of the dendrites from ventral territories into more dorsal medial domains. This was unexpected and suggests that changes in midline signalling can also impact the organization of dendrites in the dorso-ventral axis. So CommGOF 96 h AH neurons may not only encounter novel synaptic inputs by projecting into medial territories, but they may also lose inputs from the ventral domains of neuropil they have vacated. Our observations suggest that motoneurons within lineage 15 have a fixed quota of dendrites and where it is distributed in space depends on cell-intrinsic blends of guidance receptors. Taken together these data support the idea that growth and guidance mechanisms are genetically separable programs [62],[63]. In identified embryonic motoneurons where Slit-Robo and Netrin-Fra signalling has been disrupted, quantitative analysis reveals dendrites also show no measurable difference in their total number of branch tips or length [see accompanying article Mauss et al.]. Moreover, recent computational studies in larger flies reveal that dendritic arborizations generated by the same branching programs can generate very different shapes depending on how their “dendritic span” restricted within the neuropil [64]. Previous work in both vertebrates and *Drosophila* has shown that a loss of Slit-Robo signalling results in a reduction in dendrite growth and complexity [65]–[67], but we do not find evidence to support this.

### Linking Morphogenesis and Synaptic Specificity: The “Waterloo Clock” Model

Neural maps and synaptic laminae are universal features of nervous system design and are essential for organizing and presenting synaptic information. How the appropriate pre- and postsynaptic elements within such structures are brought together remains a major unanswered question in neurobiology. Studies in recent years have shown that neural network development involves both hardwired molecular guidance mechanisms and activity-dependent processes; the relative contribution that each makes is still unclear [3],[10]. Work by Li and colleagues [68] on the spinal cord network of *Xenopus* embryos revealed that seven identifiable neuron subtypes can establish connections with one another and that the key predictor of connectivity was their anatomical overlap. This could be interpreted to mean that connectivity is promiscuous and that the major requirement for the generation of synaptic specificity is the proximity of axons and dendrites. This is particularly interesting in light of our dendrite targeting data and the observation that both sensory neurons and interneurons in *Drosophila* use the same midline cues to position their pre-synaptic terminals in the CNS [24],[25]. Moreover, a recent study has shown that Semaphorins control the positioning of axons within the dorso-ventral axis [61]. Taken together these observations suggest that during development the coordinated targeting of both pre- and postsynaptic elements into the same space using global, third-party guidance signals could provide a simple way of establishing the specificity of synaptic connections within neural networks. This idea is akin to “meeting places” such as the traditional rendezvous underneath the four-sided clock at Waterloo railway station where two interested parties organize to meet. Understanding how morphogenetic programs contribute to the generation of synaptic specificity is likely to be key to solving the problem of neural network formation.

## Materials and Methods

### Fly Stocks

The following stocks were used for this study:

1. *Oregon-R*
2. *OK371-GAL4, UAS-mCD8::GFP,FRT^42B^*
3. *hsFLP, UAS-mCD8::GFP; tubGAL80, FRT^42B^/CyO*
4. *OK371-GAL4, UAS-mCD8::GFP, FRT^42B^, robo^GA2875^/CyO;+/TM6B*
5. *OK371-GAL4, UAS-mCD8::GFP, FRT^42B^, robo^ZI772^/CyO;+/TM6B*
6. *OK371-GAL4, UAS-mCD8::GFP, FRT^42B^, fra^3^/CyO;+/TM6B*
7. *OK371-GAL4, UAS-mCD8::GFP, FRT^42B^, fra^4^/CyO;+/TM6B*
8. *NetrinB-myc*
9. *FM7iGFP/FRT^19A^;Bc/CyO*
10. *FM7iGFP/FRT^19A^;+;TM3,sb/TM6B*
11. *hsFLP^122^,tub-PGAL80,FRT^19A^/FM7iGFP;OK371-GAL4,UAS-mCD8::GFP/Cyo*
12. *Sco/CyO,wglacZ;UAS-Robo::myc*
13. *UAS-HA::Robo/TM3,UbxlacZ*
14. *UAS-Fra::myc/CyO;sb/TM2*
15. *UAS-Comm::GFP*
16. *FRT^19A^;+;C161-GAL4, UAS-mCD8::GFP/TM6B*
17. *ppk-GAL4,UAS-mCD8::GFP/CyO*
18. *hsFLP;UAS-DP110*
19. *Slit-GAL4,UAS-mCD8GFP/Cyo*
20. *Slit-GAL4*
21. *UAS-mCD8GFP;;VGN9281-GAL4*
22. *UAS-Slit RNAi (VDRC Trans ID 20210)* [69]
23. *E49-GAL4*
24. *NetrinABΔ*
25. *UAS-NetrinB*

### Generation of MARCM Clones

The MARCM system [26] was used to fractionate the GAL4 enhancer trap line OK371, which strongly labels excitatory motoneurons [27]. To generate MARCM clones, eggs were collected on standard fly food for 7 h and allowed to age to the required stage (at 25°C). Following this staging heat shocks were performed by incubating vials at 37°C for 45 min, followed by a period of 30 min at 25°C and then 37°C for 30 min. Three time points were used during this study: Newly Hatched Larval stage, 48 h AH, and 96 h AH. After heatshocks, larvae were reared on standard fly food at either 25°C or room temperature when precise staging was not required.

### Immunocytochemistry

Nervous systems were dissected from larvae, pupae, and adults in phosphate buffered saline (pH 7.8) (PBS) and fixed in 3.7% buffered formaldehyde for 45 min at room temperature and then washed three times in PBS containing 1% Triton-X100 (PBS-TX). Fixed samples were blocked in PBS-TX containing 2% normal donkey serum (Jackson ImmunoResearch Laboratories, West Grove, PA, USA) for 30 min and then incubated in combinations of primary antibodies for 1 to 2 d at 4°C. After washing with PBS-TX over 8 h, tissues were incubated overnight at 4°C in combinations of secondary antibodies for 1 to 2 d at 4°C. Following repeated washes with PBS-TX, tissues were mounted on poly-L-lysine coated coverslips, dehydrated, cleared in xylene, and mounted in DPX (Fluka, Bachs, Switzerland). For the detergent free immunohistochemical method, the above protocol was repeated replacing PBS-TX with PBS at all stages.

The following primary antibodies were used: (1) rabbit (rb) anti-GFP (1∶500; Invitrogen), (2) rat (rt) anti-mCD8 (1∶500; Caltag Laboratories, Burlingame, CA, USA), (3) mouse (ms) anti-Neuroglian (BP104, 1∶5; DSHB), (4) ms anti-robo (1∶100), (5) ms anti-myc (9E 10, 1∶10; DSHB), (6) ms anti-Slit (C555.6D, 1∶300; DSHB). The following secondary antibodies were used: (1) FITC conjugated donkey anti-rabbit IgG (1∶500; Invitrogen), (2) Texas Red conjugated donkey anti-mouse IgG (1∶500; Invitrogen), (3) Alexa Fluor 488 donkey anti-Rb (1∶500; Invitrogen), and (4) FITC conjugated donkey anti-Rat IgG (1∶500; Invitrogen).

### Confocal Microscopy and Image Processing

Fluorescently stained nervous systems were imaged at 40× using a Zeiss LSM510 confocal microscope. Z-stacks were collected with optical sections at 1.5 µm intervals. Raw data stacks were imported into NIH Image J (http://rsb.info.nih.gov.nih-image/). In cases where multiple cells were labeled in nearby but non-overlapping regions, we used the lasso tool to remove any processes that obscured MN clones. In any situation where there was a possible conflict, stacks were discarded from analysis. The maximum z-projections were then imported into Photoshop (Adobe, San Jose, CA, USA) and minor adjustments were made to the brightness and contrast where required.

### Image and Data Analysis

To quantify the distribution of the dendrites of single-cell clones along the medio-lateral axis of the neuropil, we used the plot profile tool in ImageJ. Analysis was performed on maximum z-projections. Care was taken to obtain images that had equivalent grayscale ranges. A box was drawn around the whole arborization excluding the soma, the neuroglian counterstain was used for registration, and the distances along the *x*-axis were first scaled from the interval 0 (midline) to 100 (lateral edge). Plot profile displays a two-dimensional histogram of average pixel intensities along the *x*-axis. Weighted means were computed (using average pixel intensity as weights), i.e., centres of arbor mass were then used to compute statistical significance between profiles. We also computed the location of the mean 33^rd^ percentile pixel intensity to give an indication of the asymmetry of profiles. Nervous systems were measured for each experimental condition and individual profiles were overlayed using Photoshop (Adobe, San Jose, CA, USA); each histogram presents the accumulated data of 16 neurons (two groups of eight).The medio-lateral axis of the neuropil was divided into three neuropil territories: medial (M), intermediate (I), and lateral (L).

### Statistical Analysis

Statistical analysis was done with two-tailed *t* test. Using the Welch correction, *p* denotes the significance (* *p*<0.05, ** *p*<0.01, and *** *p*<0.001).

### Quantification of Dendrite Complexity

A quantitative measure of dendritic arborization complexity of individual ddaC neurons was determined using Sholl analysis [35]. The number of intersections between dendritic processes and Sholl-rings were counted in Photoshop using a template of 12 concentric circles (each 22 µm apart) centred on the cell body.

### Soma Volume Measurements

The volume of a cell was measured using Volocity Acquisition software (version 4.2, Improvision). Raw data stacks were imported and 3D projections of the cell body constructed. The lasso tool was then used to capture the cell body and the volume subsequently measured.

### Generation of VGN9281-GAL4 Line

The VGN9281-GAL4 line was generated by cloning 849 bp of wild-type upstream DNA of the *Drosophila DVGlut* gene from position 2L: 2397582, 2410668. This corresponds to from −3.8 Kb to −4.6 Kb upstream of the translation start site (used as a reference point). This region was cloned into the EcoR1 and BamH1 sites of a modified GAL4 P-element vector, pPTGAL-attB. The pPTGAL vector, a gift from Daniel F. Eberl [70], was modified by cloning 285 bp of attB sequence from pUASTB, a gift from Michele P. Calos, into the XbaI and BglII sites of pPTGAL. The *phiC31* site-specific integration system [71] was used to target the construct to chromosome 3L.

Primers used to amplify attB sequence from pUASTB were:

Forward primer,

attBFXb- 5′CAGTCTCTAGAGTCGACGATGTAGGTCACGGTC 3′ and

reverse primer,

attBRBg- 5′CAGTCAGATCTGTCGACATGCCCGCCGTGACCG 3′.

Primers used to generate VGN9281-Gal4 line were:

Forward primer,

9281EcoF-5′CAGTCGAATTCTAAGGCGATTCCTCCAAGTG 3′ and

reverse primer,

9281BamR-5′ CAGTCGGATCCGAATCGGGCGAGGACTTC 3′.

## Abbreviations

AH: after hatching
APF: after puparium formation
Comm: Commisureless
CommGOF: Comm gain of function
FraGOF: Fra gain of function
FraLOF: Frazzled loss of function
ms: mouse
PBS: phosphate buffered saline
PBS-TX: PBS containing 1% Triton-X100
RoboGOF: Robo gain of function
RoboLOF: Robo loss of function
rb: rabbit
rt: rat
